# Immunotherapy and Antiangiogenic Therapy for the Treatment of Patients With Advanced Renal Cell Carcinoma: A Systematic Review and an Updated Network Meta-Analysis of Phase III Clinical Trials

**DOI:** 10.7759/cureus.38838

**Published:** 2023-05-10

**Authors:** Rushin Patel, Afoma Onyechi, Mohamed MG Mohamed, Mosunmoluwa Oyenuga, Sara Sartaj

**Affiliations:** 1 Internal Medicine, SSM Health St. Mary's Hospital, St. Louis, USA; 2 Pulmonary and Critical Care, St. Louis University Hospital, St. Louis, USA; 3 Internal Medicine, Abbott Northwestern Hospital, Minneapolis, USA

**Keywords:** metastatic renal cell carcinoma, phase iii clinical trials, advanced renal cell carcinoma, antiangiogenic therapy, immunotherapy

## Abstract

In advanced renal cell carcinoma, few randomized controlled trials involving immunotherapy plus antiangiogenic therapy have shown survival benefits relative to Sunitinib. Our meta-analysis aimed to evaluate the efficacy and safety of combined immunotherapy and antiangiogenic therapy compared to Sunitinib therapy alone in patients with advanced renal cell carcinoma. Six phase III randomized controlled trials were analyzed, including 4,119 patients. The primary endpoints were overall survival and progression-free survival, and the secondary endpoints were objective response rate and serious adverse events. The results showed that combined immunotherapy and antiangiogenic therapy significantly improved overall survival, progression-free survival, and objective response rate compared to Sunitinib alone. No significant difference was observed in adverse events between the two groups. This study suggests that combined immunotherapy and antiangiogenic therapy is a great treatment option for advanced renal cell carcinoma.

## Introduction and background

Renal cell carcinoma (RCC) arises from the renal parenchymal urinary tubule epithelial system. It is the most prevalent renal neoplasm and nearly 30% of patients have metastatic disease, which increases RCC mortality [1]. It is only second in incidence to the bladder. The incidence of RCC increases significantly with age, affecting men more than women.

RCC is not sensitive to radio-chemotherapy, and the five-year survival rate is less than 50% in advanced RCC [2]. The main causes of relapse after RCC treatment and the key factors impacting patient prognosis are lymph node metastasis and cancer cell infiltration [2]. Historically, due to their typical resistance to conventional chemotherapies, radiotherapies, and hormone treatments, advanced RCC has a poor prognosis. Notably, in recent times, the treatment landscape has changed dramatically, with many new therapeutic options and improved patient survival. Novel treatments include combination therapies using molecularly targeted agents against the vascular endothelial growth factor (VEGF) pathway, as well as immune checkpoint inhibitors (ICIs), which stimulate an anti-tumor immune response [3].

The formation of new blood vessels is fundamental for tumor development, and RCC is well known for high vascularization. Consequently, several methods used to treat advanced RCC are designed to prevent angiogenesis. For instance, a hypoxia-inducible factor is degraded by a pathway that involves Von Hippel-Lindau (VHL), which is a tumor suppressor gene. Mutation of VHL frequently occurs in RCC, resulting in the transcription of numerous genes, including VEGF, which promotes angiogenesis [3]. Lately, management of RCC has centered on utilizing targeted agents to block the VEGF pathway and other receptors involved in angiogenesis [3].

RCC is also a highly immunogenic tumor and it has been demonstrated that T-cells, dendritic cells, macrophages, and natural killer cells are present in RCC tumors as an immune cell infiltration. T-cells have a protein on their surface called “programmed cell death-1” (PD-1) that, when attached to “programmed death ligand-1,” suppresses an immune response (PD-L1). Both PD-L1 and PD-L2 are PD-1 ligands, and malignant cells typically express PD-L1 which is generally found in antigen-presenting cells. ICIs disrupt the PD-1/PD-L1 axis, restore the function of effector T cells, and decrease the activity of regulatory T-cells, thereby fostering an anti-tumor immune [4].

Tumor immunology has advanced significantly over the past years, resulting in the creation of ICIs that have been demonstrated to boost antitumor immunity in a variety of cancer types, including RCC [4]. Studies have shown a potential synergistic effect of both antiangiogenic therapy and immunotherapy for advanced RCC because when combined, this treatment modality targets these tumor features in separate ways [4]. We, therefore, performed an updated meta-analysis of randomized controlled trials (RCTs) to evaluate the efficacy of immunotherapy plus antiangiogenic therapy in patients with advanced RCC compared to Sunitinib therapy alone.

## Review

Materials and methods

Search Strategy and Data Extraction

A comprehensive search was conducted across several databases, including PubMed, Cochrane Library, Google Scholar, Scopus, and Clinical trial.gov, covering all available literature up to September 1, 2022 using pre-specified search terms, “Immunotherapy with antiangiogenic therapy” OR “immunotherapy in addition to antiangiogenic therapy” OR “antiangiogenic therapy added to immunotherapy” AND “advanced renal cell carcinoma” OR “metastatic renal cell carcinoma” OR “inoperable renal cell carcinoma” AND “Sunitinib” OR “Sutent”. The search was limited to the English language.

Inclusion/exclusion criteria

This analysis was limited to prospective phase 3 RCTs that compared any combination of immunotherapy with antiangiogenic therapy to Sunitinib in patients aged 18 or above, diagnosed with RCC. Only studies that defined their patients as having advanced, recurrent, unresectable, or inoperable diseases were included. Furthermore, to be eligible for this analysis, studies had to report at least the primary outcome. Trials that lacked an active comparator arm, non-randomized trials, and observational studies were excluded from the analysis.

Endpoint/outcomes

Co-primary outcomes of interest were overall survival (OS) and disease/progression-free survival (PFS) as defined per respective studies. Secondary efficacy outcome was objective response rate (as defined per each respective study). We also reported safety outcomes in terms of serious adverse events (Grade 3 or more). (Grade 3 adverse event defined as severe or medically significant but not immediately life-threatening; hospitalization or prolongation of hospitalization indicated; disabling; limiting self-care ADL.)

Statistical Analysis

For dichotomous data, odds ratios (OR) were calculated, and for survival data, generic inverse variance with hazard ratios (HR) and their corresponding 95% confidence intervals (CI) were computed. Heterogeneity was reported using I2 statistics. The analysis was performed using RevMan 5.4 software, accounting for the weight of different trials.

Search Results and Study Selection

**Figure 1:**
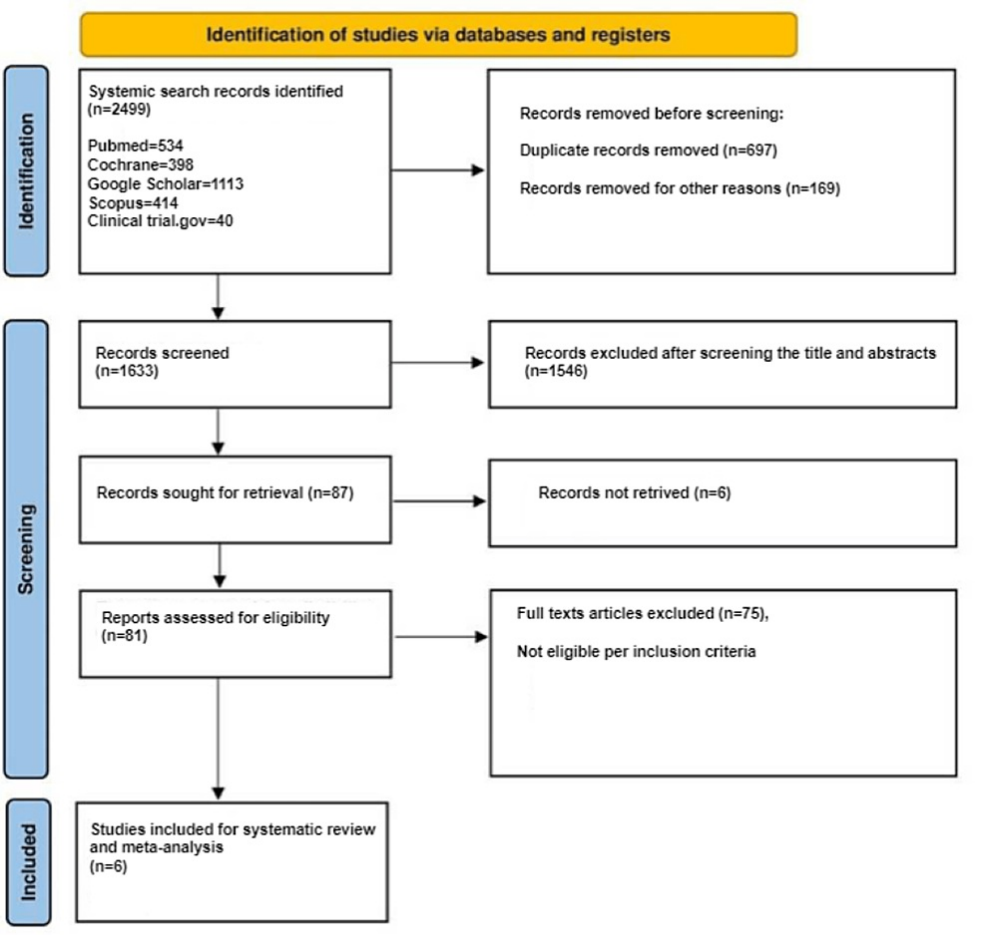
PRISMA flow diagram detailing the study selection process

A total of 2,499 studies were initially retrieved through the literature search, out of which 1,633 underwent abstract and title screening after removing duplicates. Following this, 87 studies were identified for full-text review, and the primary reason for exclusion was not fulfilling the eligibility criteria. Eventually, six trials were included in the systematic review and meta-analysis. The PRISMA flow diagram in the figure summarizes the search results and the study selection process based on PRISMA guidelines (Figure 1) [5].

Trials Included

Six studies were eligible [6-11], with a total of 4,119 patients (immunotherapy + antiangiogenic therapy=2,050, Sunitinib=2,069), and a mean follow-up of ~27 months (Table 1).

**Table 1:** Outcomes of the phase 3 clinical trials included in meta-analysis E = Exposure group (Immunotherapy+ antiangiogenic therapy), C = Control group (Sunitinib)

	Keynote 426 [6]	Keynote 426 post-hoc analysis subgroup [7]	Keynote 581 [8]	Updated Checkmate 9ER [9]	Updated IMmotion 151 [10]	Updated Javelin renal 101 [11]
Population studied	Previously untreated advanced RCC	Previously untreated advanced RCC	Advanced RCC	Previously untreated advanced RCC	Metastatic RCC	Advanced RCC
Treatment groups	Pembrolizumab plus Axitinib versus Sunitinib	Pembrolizumab plus Axitinib versus Sunitinib	Pembrolizumab plus Lenvatinib versus Sunitinib	Nivolumab plus Cabozantinib versus Sunitinib	Atezolizumab plus Bevacizumab versus Sunitinib	Avelumab plus Axitinib versus sunitinib
Number of participants	E=432 C=429	E=44 C=50	E=355 C=357	E=35 C=357	E=454 C=461	E=442 C=444
Median follow-up	12.8 months	29.5 months	26.6 months	32.9 months	40 months	19 months
Hazard ratio for death (Overall surival)	HR= 0.53 (95% CI, 0.38–0.74) P<0.0001	HR=0.83(95% Cl, 0.39-1.76) P=0.62	HR=0.66 (95% CI,0.49–0.88) P=0.005	HR=0.70 (95% CI, 0·55–0·90) P=0·0043	HR= 0.81 (95% CI, 0.63 to 1.03) P= 0.0895	HR=0.80 (95% CI, 0.62–1.03) P=0.039
Hazard ratio for disease progression or death ( progression free survival)	HR 0.69 (95% CI, 0.57-0.84); P<0.001	HR= 0.61 (95% Cl, 0.37-1.01) P=0.054	HR= 0.39 (95% CI, 0.32–0.49) P<0·0001	HR=0.56 (95% CI, 0·46−0·68) P<0·0001	HR= 0.73(95% CI, 0.57 to 0.95) P = 0.0205	HR=0.69 (95% CI, 0.57–0.83) P< 0.0001
Objective response rate	E=256 C=153	E=31 C=26	E= 252 C=129	E=180 C=89	E=168 C=152	E=232 C=121
Grade 3 or higher adverse events	E=327 C=303	E=31 C=38	E= 290 C=244	E=208 C=172	E=205 C=250	E=309 C=314

Results

**Figure 2:**
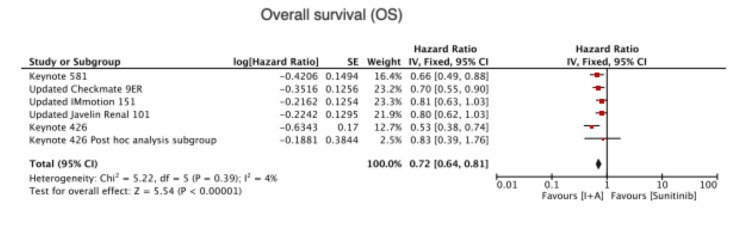
Overall survival Keynote 426 [6], Keynote 426 post-hoc analysis subgroup [7], Keynote 581 [8], Updated Checkmate 9ER [9], Updated IMmotion 151 [10], Updated Javelin renal 101 [11]

Compared to Sunitinib alone, combined immunotherapy and antiangiogenic therapy showed significantly higher overall survival (HR=0.72 (0.64,0.81), P < 0.00001) (Figure 2).

**Figure 3:**
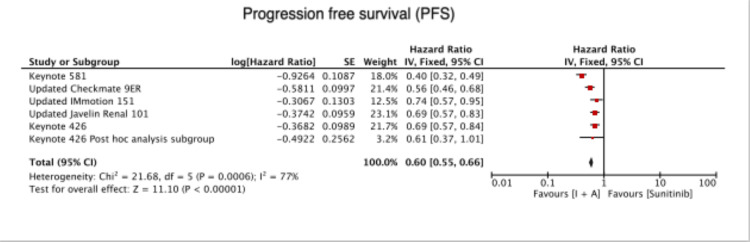
Progression free survival Keynote 426 [6], Keynote 426 post-hoc analysis subgroup [7], Keynote 581 [8], Updated Checkmate 9ER [9], Updated IMmotion 151 [10], Updated Javelin renal 101 [11]

Compared to Sunitinib alone, combined immunotherapy and antiangiogenic therapy showed significantly higher PFS (HR=0.60 [0.55, 0.66), P < 0.00001) (Figure 3).

**Figure 4:**
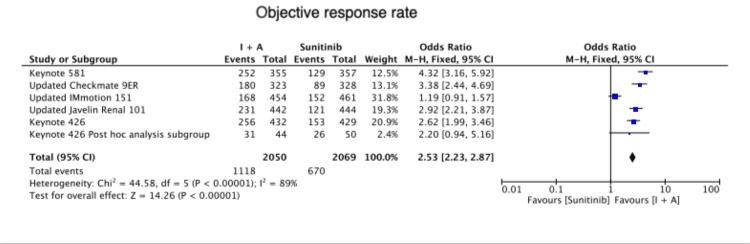
Objective response rate Keynote 426 [6], Keynote 426 post-hoc analysis subgroup [7], Keynote 581 [8], Updated Checkmate 9ER [9], Updated IMmotion 151 [10], Updated Javelin renal 101 [11]

Combined immunotherapy and antiangiogenic therapy also showed a favorable trend compared to Sunitinib regarding objective response rate (OR=2.53 (2.23, 2.87), P < 0.00001) (Figure 4).

**Figure 5:**
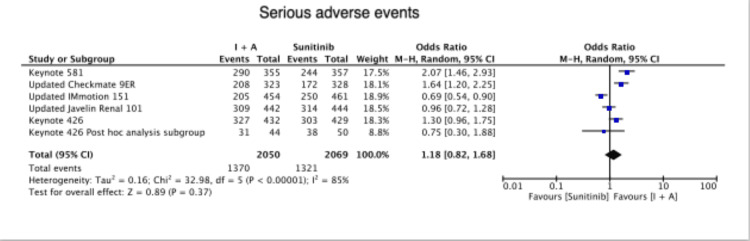
Serious adverse events Keynote 426 [6], Keynote 426 post-hoc analysis subgroup [7], Keynote 581 [8], Updated Checkmate 9ER [9], Updated IMmotion 151 [10], Updated Javelin renal 101 [11]

There was no difference in serious adverse events between the two groups (OR=1.18 (0.82,1.68), P = 0.37) (Figure 5).

Discussion

Our meta-analysis showed that patients with advanced RCC who were treated with combined immunotherapy and antiangiogenic therapy had a higher overall survival and PFS compared with those treated with Sunitinib alone. There was also a favorable trend with the objective response. No significant difference was observed in adverse events between the two groups.

Phase III open label KEYNOTE trial 426 showed treatment with pembrolizumab and axitinib resulted in a higher objective response with overall longer survival and a longer PFS [12]. In 2021, Motzer et al evaluated the efficacy of lenvatinib with pembrolizumab and lenvatinib with everolimus in comparison with sunitinib alone. They concluded that the combination of lenvatinib and pembrolizumab had a longer PFS and OS per se. However, treatment with lenvatinib and everolimus did not result in a significantly greater difference in overall survival [8]. Trial IMmotion151 compared atezolizumab plus bevacizumab versus sunitinib in metastatic RCC and depicted prolonged PFS when compared to sunitinib after an extended follow up [10]. In the second interim analysis of the JAVELIN renal 101 trial which compared first-line avelumab plus axitinib versus sunitinib, avelumab plus axitinib continued to show a prolonged PFS [11].

Commonly reported adverse effects are hepatotoxicity with pembrolizumab, thyroid abnormalities with axitinib use [12]. Adverse effects of palmar plantar erythro-dysesthesia, fatigue, and loss of appetite were reported more in sunitinib when compared to Atezolizumab plus bevacizumab [10]. Our meta-analysis showed no difference in adverse effects profile between the combined immunotherapy and antiangiogenic therapy versus sunitinib though in a recent meta-analysis, the use of the combined therapy was associated with higher risk of all grade adverse effects including grade 3 and higher for diarrhea, increased appetite, and increased aspartate and alanine transaminase [13].

The major strength of our meta-analysis lies in our use of phase III trials which compared the use of combined immunotherapy and antiangiogenic therapy with the current standard of care. It also showed a better safety profile when immunotherapy was combined with antiangiogenic therapy. However, we only compared the findings of a limited number of trials. Another limitation would be the shorter duration of the follow up in some of the trials which were analyzed in our meta-analysis. Although cross-trial comparisons are difficult to make, dual pathway targeting seems to be an effective strategy in the treatment of advanced renal cell cancers [11].

## Conclusions

In summary, it can be concluded that the combination therapy of immunotherapy and antiangiogenic therapy results in higher overall survival, PFS, and objective response rates, without any significant increase in adverse events when compared to the use of Sunitinib alone. This meta-analysis provides strong evidence for the potential of combining these therapies, but it should be noted that the long-term effectiveness of this approach still requires further investigation with extended follow-up periods. Despite this, the results of this study suggest that this treatment strategy may have significant benefits for patients with advanced RCC, and it is hoped that further research in this area will be conducted in the future.
